# Effects on Outcomes of Hyperglycemia in the Hyperacute Stage after Acute Traumatic Spinal Cord Injury

**DOI:** 10.1089/neur.2020.0042

**Published:** 2021-01-19

**Authors:** Julio C. Furlan

**Affiliations:** ^1^ Department of Medicine, Division of Physical Medicine and Rehabilitation, University of Toronto, Toronto, Ontario, Canada.; ^2^ Rehabilitation Sciences Institute, University of Toronto, Toronto, Ontario, Canada.; ^3^ Lyndhurst Centre, KITE - Toronto Rehabilitation Institute, University Health Network, Toronto, Ontario, Canada.

**Keywords:** disability, hyperglycemia, neurological impairment, spinal cord injury, survival

## Abstract

Hyperglycemia has adverse effects on neuronal recovery after brain injury, but its effects after spinal cord injury (SCI) are understudied. This retrospective cohort study examined the potential effects on outcomes of hyperglycemia in the hyperacute stage after acute traumatic SCI. This study included all individuals enrolled in the National Acute Spinal Cord Injury Study 3 (NASCIS-3). Glycemic levels at 24 h, at 48 h, and at day 7 after acute SCI were examined as potential determinants of survival, neurological outcomes (using NASCIS motor, sensory, and pain scores), and functional outcome (using the Functional Independence Measure [FIM]) within the first year post-SCI. Hyperglycemia was defined using two thresholds (140 mg/dL and 180 mg/dL). Study subjects were 76 females and 423 males with an overall mean age of 36 years who sustained mostly cervical SCI due to motor vehicle accidents or falls. Hyperglycemia diagnosed at day 7 post-injury was associated with significantly greater mortality rates post-SCI. Among the survivors, hyperglycemia during the hyperacute stage was not significantly correlated with neurological recovery post-SCI. Hyperglycemia persistent until day 7 was significantly correlated with lower functional scores post-SCI. These results suggest that hyperglycemia at day 7 is correlated with greater mortality rates within the first year post-SCI. Although hyperglycemia during the hyperacute stage was not associated with neurological recovery, hyperglycemia at day 7 may adversely affect functional recovery within the first year post-SCI. Future investigations are needed to determine the optimal glycemic target in the management of patients with SCI.

## Introduction

Traumatic spinal cord injury (SCI) remains an important cause of disability due to motor, sensory, and autonomic impairment that has a significant worldwide health impact on individuals and society. The worldwide incidence rate of traumatic SCI varies from 8 to 246 cases per million inhabitants per year, and in the literature its global prevalence rate ranges from 236 to 1298 per million inhabitants per year.^1^ Promising scientific discoveries on neuroprotective and cell-based therapies for SCI have not yet been translated into clinical practice, and currently the best outcomes from the management of individuals with acute traumatic SCI rely on recent advancements in pre-hospital care, acute care, and rehabilitation.^2–4^ In the acute care setting, early surgical decompression of the spinal cord and maintenance of spinal cord pressure have become best practice in the management of patients with acute traumatic SCI.^5–8^ Accordingly, a better understanding of other predictors of outcomes after injury is of the utmost importance to further improve the initial care of patients with acute traumatic SCI.

The potential effects of hyperglycemia after neurotrauma have been studied primarily in patients with traumatic brain injury (TBI). Pre-clinical and clinical evidence indicate that hyperglycemia and hypoglycemia can adversely affect neurological recovery after TBI.^9^ This has led clinicians to promote vigilant adjustment of hyperglycemia during the management of patients in the hyperacute stage following TBI.^9^ The same clinical approach has been applied in many acute spine centers in the management of individuals with acute traumatic SCI, even though most of the current evidence for this approach has actually been transferred from knowledge on managing individuals with TBI.

The etiology of hyperglycemia after central nervous system (CNS) injury is multi-factorial.^10^ First, stress-induced hyperglycemia is considered an adaptive immune-neurohormonal response to stress involving two mechanisms: 1) enhanced glycogenolysis and hypermetabolism induced by secretion of stress hormones such as catecholamine, cortisol, glucagon, and growth hormone after activation of the hypothalamic-pituitary-adrenal axis and the sympathetic autonomic nervous system; and 2) transient increase in insulin resistance as another catecholamine-related effect.^10^

Second, both TBI and SCI are often accompanied by a systemic inflammatory response syndrome that can cause hyperglycemia due to: 1) release of several cytokines (e.g., tumor necrosis factor alpha [TNF-α], interleukin [IL]-6, and CD11d) provoking hyperglycemia and insulin resistance; and 2) release of the corticotrophin-releasing hormone and release of adrenocorticotropic hormone from the anterior pituitary.^10^

Third, pre-injury diabetes mellitus, which had an estimated prevalence of 4.9% in the United States in 1990 and an estimated global prevalence of 9.3% in 2019, is another potential cause of hyperglycemia in individuals with neurotrauma.^10,11^ Fourth, pituitary and hypothalamic dysfunction has multiple effects on the stress response, and directly affects glucose homeostasis, hepatic gluconeogenesis, and insulin sensitivity.^10^

Finally, many other factors inherent to the management of individuals with an acute CNS injury can incite hyperglycemia including fluid infusions, surgery, anesthesia, and drugs (e.g., corticosteroids).^10^

With this background, a retrospective cohort study was carried out using a large cohort of individuals with SCI to examine whether hyperglycemia during the hyperacute stage post-injury is associated with the individuals' survival, and their neurological and functional recovery within the first year following traumatic SCI.

## Methods

The Institutional Ethics Board approved the use of the database from the National Acute Spinal Cord Injury Study 3 (NASCIS-3) to address research questions other than the original aims of that trial. A Data Use Agreement between Yale University and the University Health Network was instituted in 2006.

This retrospective cohort study included all 499 individuals who were enrolled in the NASCIS-3. Of note, that trial was designed to compare a 48-h methylprednisolone (MPSS) protocol with 24-h MPSS administration in the management of patients with acute traumatic SCI.^12^ The NASCIS-3 also included a third study arm in which individuals received 48-h intravenous administration of tirilazad mesylate (also known as Lazaroids), a non-glucocorticoid potent lipid peroxidation inhibitor that showed neuroprotective properties in pre-clinical studies.^12^ All participants in the NASCIS-3 were randomized within 6 h from the time of SCI to receive intravenous MPSS for 24 h or 48 h, or intravenous tirilazad mesylate for 48 h. Treatment was initiated within 8 h from SCI onset.^12^ Individuals age 14 years or older with acute traumatic SCI were considered eligible to be recruited into the NASCIS-3. Complete inclusion and exclusion criteria are documented elsewhere.^12^

### Outcome measures

Survival data within the first year after SCI were analyzed in this study. In all enrolled individuals, the degree of impairment was assessed using the NASCIS motor, sensory, and pain scores at admission to the emergency department as well as at 6 weeks, at 6 months, and at 1 year following SCI. The NASCIS motor score was determined by assessing motor function in 14 segments bilaterally, using a manual muscle scoring system as follows: 0 for no muscle contraction; 1 for trace of muscle contraction; 2 for active movement without antigravity; 3 for active movement with antigravity; 4 for active movement against resistance; and 5 for normal function. The NASCIS motor scores ranged from 0 (no contraction in any muscle) to 70 (all normal motor responses).^12^ The NASCIS sensory score was determined by testing pinprick and light touch sensation in each dermatome from vertebral level C2 to S5 bilaterally. Each sensory response was classified as: 1 if absent, 2 if dysfunctional (including hypoesthesia and hyperesthesia), or 3 if normal response. Each sensory score ranged from 29 (all absent sensory responses) to 87 (all normal sensory responses).^12^ The NASCIS pain score was bilaterally established according to the responses to deep pain and pressure at the wrist, thumb, little finger, knee, ankle, and great toe areas. Each response to deep pain and pressure was classified as: 1 for absent, 2 for decreased, and 3 for normal response. The NASCIS pain score ranged from 6 (all absent pain and pressure responses) to 18 (all normal responses).

The degree of disability was assessed using the Functional Independence Measure (FIM) score.^13^ The FIM score includes sub-scores for self-care, sphincter control, mobility, locomotion, communication, and social cognition. The FIM score ranged from 18 (need for assistance in all areas) to 126 (complete independence). All individuals were assessed for degree of disability at 6 weeks, at 6 months, and at 1 year following SCI.

### Laboratory blood tests

According to NASCIS-3 protocol, blood samples for glycemic level and serum creatinine concentration were drawn at 24 h, at 48 h, and at day 7 after SCI onset from all participants. Normal reference values in glycemic testing range from 80 to 140 mg/dL. Given the variability in the literature of the definition of hyperglycemia, all data analyses in this study used the lower and upper thresholds of most commonly accepted definitions for “stress-induced hyperglycemia” as follows: 1) a random glycemic test result greater than 140 mg/dL; and 2) a random glycemic test result greater than 180 mg/dL.^14,15^

### Statistical analysis

Analysis of variance (ANOVA) with Bonferroni post hoc test was used to compare the results of glycemic tests within the first week after SCI using continuous variables. The time effect on glycemic levels was evaluated using repeated measures ANOVA. Survival analysis was carried out using Kaplan-Meier curve and log-rank test.

Multiple regression analyses were used to examine within the first week after SCI the potential effects of hyperglycemia on the neurological outcomes (i.e., NASCIS motor, sensory, and pain scores). Those multiple regression analyses were adjusted for the respective neurological status at admission, individuals' sex and age at SCI onset, NASCIS-3 trial drug protocol (i.e., MPSS for 24 h, MPSS for 48 h, or tirilazad mesylate for 48 h), level of SCI (i.e., cervical, thoracic, or lumbosacral), Glasgow Coma Scale (GCS) score on admission, and serum creatinine concentration (which was collected at the same time as glycemic testing). Similarly, multiple regression analyses were used to examine within the first week after SCI the potential effects of hyperglycemia on the degree of disability as assessed using the FIM at 6 weeks, at 6 months, and 1 year after SCI. Those multiple regression analyses used for FIM scores as the dependent variable were adjusted for the degree of motor impairment on admission (as assessed using the NASCIS motor score), individuals' sex and age at the SCI onset, NASCIS-3 trial drug protocol (i.e., MPSS for 24 h or 48 h, or tirilazad mesylate for 48 h), level of SCI (i.e., cervical, thoracic, or lumbosacral), GCS score on admission, and serum creatinine concentration (which was collected at the same time as glycemic testing). Significance was assumed at *p* < 0.05. All data analyses were carried out using SAS software, version 9.4.

## Results

Of the 499 individuals enrolled in the NASCIS-3, there were 76 females and 423 males with an overall mean age of 36 years (range, 14–92 years) who mostly sustained cervical SCI due to motor vehicle accident followed by falls (Table 1). The maximum glycemic level among the individuals with hyperglycemia was 533 mg/L. There was a significant decline in the initial mean glycemic levels within 24 h (188.20 ± 2.29 mg/dL) when compared with the mean glycemic levels at 48 h (164.44 ± 2.08 mg/dL) and at day 7 after SCI (125.02 ± 2.25 mg/dL; *p* < 0.0001). Using repeated measures ANOVA, there was a statistically significant effect of time on glycemic levels, *F* (2, 784) = 324, *p* < 0.0001.

**Table 1.** Baseline Data on the Study Cohort (*n* = 499)

Characteristics	Results
Mean age ± SEM	35.7 ± 0.76 years
Age range	14–92 years
Sex:	
Females	15.23%
Males	84.77%
Ethnicity	
Non-Hispanic white	75.35%
African American	12.02%
Hispanic	7.62%
Other ethnic groups	5.01%
Level of SCI:	
Cervical SCI	67.88%
Thoracolumbar SCI	32.12%
Extent of SCI:	
Complete SCI	49.90%
Incomplete SCI	42.45%
Spine trauma with minor deficit	7.65%
Cause of SCI:	
Motor vehicle accident	36.47%
Falls	27.25%
Motorcycle accident	8.22%
Water-related accident	8.22%
Crush injury	7.82%
Other causes	12.02%
Mean NASCIS motor score (at admission) ± SEM	60.58 ± 1.86
NASCIS motor score (at admission) range	0–140
Mean NASCIS sensory score (at admission) ± SEM	240.45 ± 3.27
NASCIS sensory score (at admission) range	116–340
Mean NASCIS pain score (at admission) ± SEM	24.80 ± 0.39
NASCIS pain score (at admission) range	12–36
Mean Glasgow Coma Scale Score ± SEM	14.57 ± 0.05
Glasgow Coma Scale score range	10–15
Mean serum alcohol level ± SEM	0.05 ± 0.01 ‰
Serum alcohol level range	0–1.00 ‰
Treatment protocols for the NASCIS-3:	
Intravenous methylprednisolone for 24 h	33.27%
Intravenous methylprednisolone for 48 h	33.27%
Intravenous tirilazadmesylate for 48 h	33.47%

NASCIS, National Acute Spinal Cord Injury Study; SCI, spinal cord injury; SEM, standard error of mean.

**FIG. 1.**
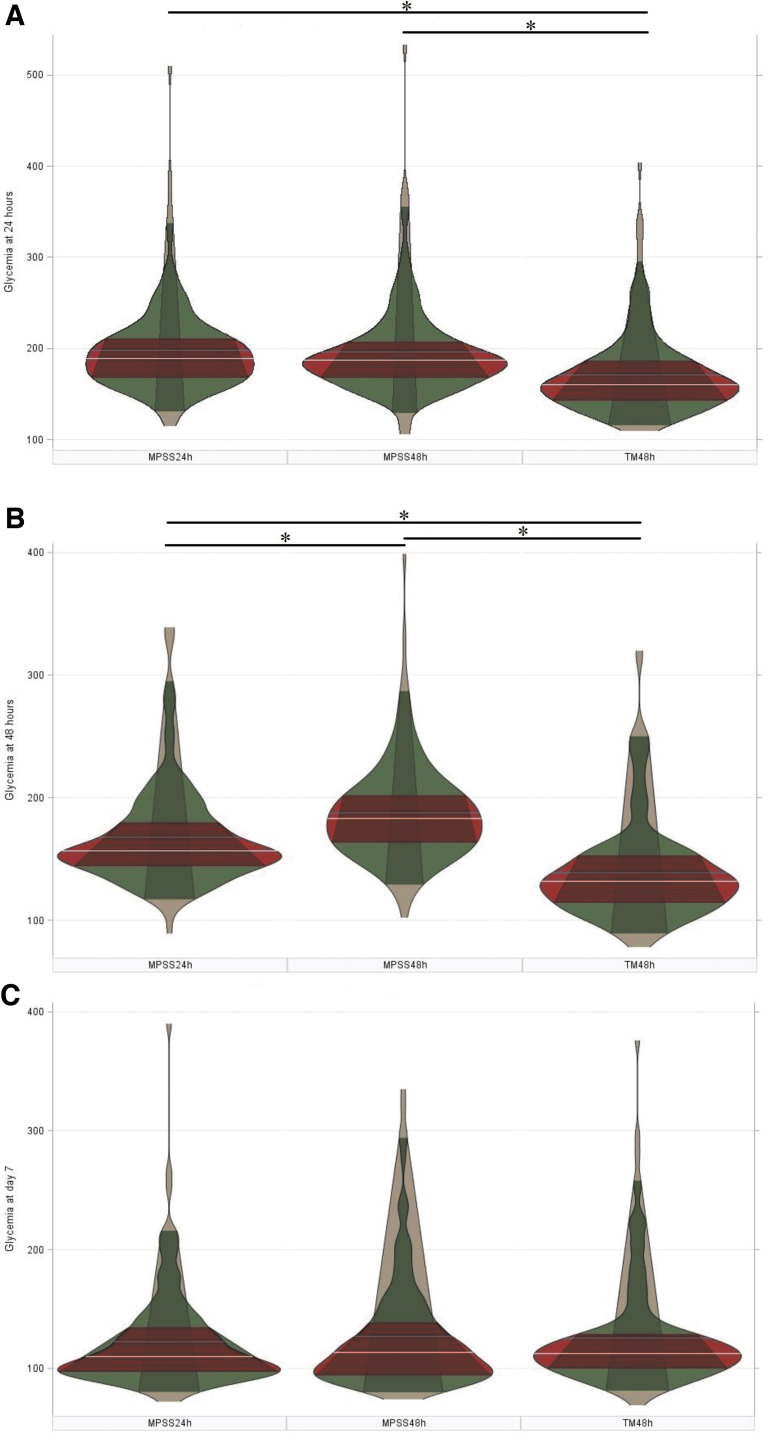
Comparisons among the groups of patients who received tirilazad mesylate for 48 hours (48-hr TM), methylprednisolone for 24 hours (24-hr MPSS) or methylprednisolone for 48 hours (48-hr MPSS) after onset of traumatic spinal cord injury (SCI). Asterisks identify statistically significant differences based on ANOVA with Bonferroni *post hoc* test.

Mean glycemic levels at 24 h post-injury were significantly lower in the group of patients who received tirilazad mesylate for 48 h than in the groups of patients who received either MPSS for 24 h or MPSS for 48 h (F value = 9.18; R^2^ = 0.0374; *p* = 0.0001; Fig. 1A). Mean glycemic levels at 48 h significantly differed among the three groups of patients (F value = 27.69; R^2^ = 0.1056; *p* < 0.0001; Fig. 1B). However, mean glycemic levels at day 7 post-injury were statistically comparable among the group of patients who received tirilazad mesylate for 48 h, the group of patients who received MPSS for 24 h, and the group of patients who received MPSS for 48 h (F value = 0.81; R^2^ = 0.0040; *p* = 0.4444; Fig. 1C).

### Hyperglycemia defined as a blood glucose level >140 mg/dL

**FIG. 2.**
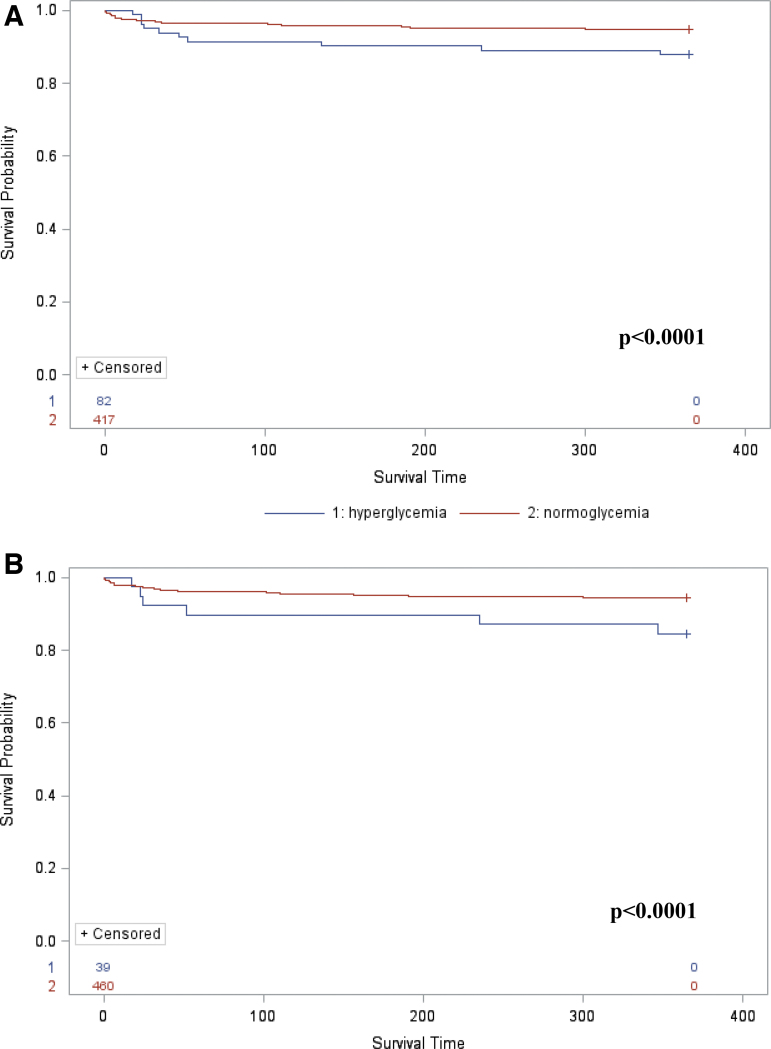
Survival analysis comparing individuals with hyperglycemia at day 7 after acute traumatic spinal cord injury, using Kaplan-Meier curve with log-rank test. **(A)** Hyperglycemia was defined as a glycemic level higher than 140 mg/dL. **(B)** Hyperglycemia was defined as a glycemic level higher than 180 mg/dL.

The majority of the individuals (78.76%) presented hyperglycemia (defined as a random glycemic level >140 mg/dL) within 24 h after the SCI onset. Hyperglycemia either at 24 h or at 48 h post-injury was not associated with mortality rates within the first year after SCI (*p* = 0.8258 and *p* = 0.8727, respectively). Nevertheless, hyperglycemia at day 7 post-injury was significantly associated with a greater mortality rate within the first year after SCI (*p* < 0.0001; Fig. 2A).

Among the survivors, the results of the multiple regression analyses indicated that hyperglycemia at 24 h, at 48 h, or at day 7 post-injury was not significantly associated with motor, sensory, or pain scores at 6 weeks, at 6 months, and at 1 year following acute traumatic SCI (Table 2; Supplementary Tables SA1–SA3). Although hyperglycemia at 24 h post-injury was not significantly associated with FIM score at 6 weeks following SCI (*p* = 0.4162), hyperglycemia at 24 h post-injury was significantly correlated with higher FIM scores at 1 year after SCI (*p* = 0.0225). There was also a trend toward an association between hyperglycemia at 24 h post-injury and higher FIM scores at 6 months after SCI (*p* = 0.0547) (Supplementary Table SA4). In contrast, hyperglycemia at day 7 post-injury was significantly associated with lower FIM scores at 6 weeks (*p* = 0.0059), at 6 months (*p* = 0.0020), and at 1 year (*p* = 0.0048) following SCI (Table 2; Supplementary Table SA4).

**Table 2.** Summary of the Multiple Regression Analyses on the Potential Effects of Hyperglycemia after SCI (Threshold >140 mg/dL)

Outcomes following SCI	Hyperglycemia at 24 h	Hyperglycemia at 48 h	Hyperglycemia at day 7
Motor score at 6 weeks	No significant effect	No significant effect	No significant effect
Sensory score at 6 weeks	No significant effect	No significant effect	No significant effect
Pain score at 6 weeks	No significant effect	No significant effect	No significant effect
FIM score at 6 weeks	No significant effect	No significant effect	No significant effect
Motor score at 6 months	No significant effect	No significant effect	No significant effect
Sensory score at 6 months	No significant effect	No significant effect	No significant effect
Pain score at 6 months	No significant effect	No significant effect	No significant effect
FIM score at 6 months	Higher functional scores	No significant effect	Lower functional scores
Motor score at 1 year	No significant effect	No significant effect	No significant effect
Sensory score at 1 year	No significant effect	No significant effect	No significant effect
Pain score at 1 year	No significant effect	No significant effect	No significant effect
FIM score at 1 year	Higher functional scores	No significant effect	Lower functional scores

At 24 h, at 48 h, or at day 7 after SCI onset on either degree of impairment (as assessed using NASCIS motor, sensory, or pain scores) or degree of disability (as assessed using FIM), after adjusting model for the respective neurological status (or NASCIS motor score at admission when using FIM score the dependent variable), individuals' age and sex, severity and level of SCI, NASCIS-3 drug protocol, Glasgow Coma Scale score on admission, and serum creatinine concentration at the same time as the blood draw for the glycemic test.

FIM, Functional Independence Measure; NASCIS, National Acute Spinal Cord Injury Study; SCI, spinal cord injury.

### Hyperglycemia defined as a blood glucose level >180 mg/dL

Fewer individuals (43.69%) presented hyperglycemia (defined as a random glycemic level >180 mg/dL) within 24 h after the SCI onset. Hyperglycemia either at 24 h or at 48 h post-injury was not associated with mortality rates within the first year after SCI (*p* = 0.3662 and *p* = 0.4113, respectively). However, hyperglycemia at day 7 post-injury was significantly correlated with higher mortality rate within the first year after SCI (*p* < 0.0001; Fig. 2B).

Among the survivors, the results of the multiple regression analyses indicated that hyperglycemia at 24 h, at 48 h, or at day 7 post-injury was not significantly associated with motor, sensory, or pain scores at 6 weeks, at 6 months, or at 1 year following acute traumatic SCI (Table 3; Supplementary Tables SB1–SB3). Further, hyperglycemia at 24 h, at 48 h, or day 7 post-injury was not significantly associated with FIM scores at 6 weeks, at 6 months, and at 1 year after SCI. However, there was a trend toward a correlation between hyperglycemia at day 7 post-injury and lower FIM scores at 6 weeks after SCI (*p* = 0.0505; Table 3; Supplementary Table SB4).

**Table 3.** Summary of the Multiple Regression Analyses on the Potential Effects of Hyperglycemia (Threshold >180 mg/dL)

Outcomes following SCI	Hyperglycemia at 24 h	Hyperglycemia at 48 h	Hyperglycemia at day 7
Motor score at 6 weeks	No significant effect	No significant effect	No significant effect
Sensory score at 6 weeks	No significant effect	No significant effect	No significant effect
Pain score at 6 weeks	No significant effect	No significant effect	No significant effect
FIM score at 6 weeks	No significant effect	No significant effect	Lower functional scores
Motor score at 6 months	No significant effect	No significant effect	No significant effect
Sensory score at 6 months	No significant effect	No significant effect	No significant effect
Pain score at 6 months	No significant effect	No significant effect	No significant effect
FIM score at 6 months	No significant effect	No significant effect	No significant effect
Motor score at 1 year	No significant effect	No significant effect	No significant effect
Sensory score at 1 year	No significant effect	No significant effect	No significant effect
Pain score at 1 year	No significant effect	No significant effect	No significant effect
FIM score at 1 year	No significant effect	No significant effect	No significant effect

At 24 h, at 48 h, or at day 7 after SCI onset on either degree of impairment (as assessed using NASCIS motor, sensory, or pain scores) or degree of disability (as assessed using FIM), after adjusting the model for the respective neurological status (or NASCIS motor score at admission when using FIM score the dependent variable), individuals' age and sex, severity and level of SCI, NASCIS-3 drug protocol, Glasgow Coma Scale score on admission, and serum creatinine concentration at the same time as the blood draw for the glycemic test.

FIM, Functional Independence Measure; NASCIS, National Acute Spinal Cord Injury Study; SCI, spinal cord injury.

## Discussion

The results of this retrospective cohort study indicate that glycemic levels frequently raise during the hyperacute stage after traumatic SCI. Although administration of MPSS was associated with increased mean glycemic levels during the period of glucocorticoid therapy, the mean glycemic levels at day 7 post-injury in the groups of patients who received MPSS did not significantly differ from those of the group of patients who received tirilazad mesylate. Hyperglycemia at 24 h or 48 h post-injury was not significantly associated with survival rates within the first year after acute traumatic SCI. However, hyperglycemia at day 7 post-injury was associated with significantly greater mortality rates within the first year after acute traumatic SCI irrespective of the threshold used to define hyperglycemia. Among the survivors, hyperglycemia at 24 h, at 48 h, and at day 7 post-injury did not adversely affect neurological recovery at 6 weeks, at 6 months, or at 1 year following acute traumatic SCI regardless of the threshold used to define hyperglycemia. Hyperglycemia at 24 h was significantly associated with higher FIM scores in the chronic stage after SCI, whereas persistent hyperglycemia at day 7 was significantly correlated with lower FIM scores at 6 weeks, at 6 months, and at 1 year after acute traumatic SCI, when a threshold of 140 mg/L was used to define hyperglycemia.

### Hyperglycemia and survival after CNS injury

The results of this study suggest that hyperglycemia within the initial 48 h may not influence survival after SCI, but persistent hyperglycemia at day 7 post-injury is significantly associated with reduced survival rates within the first year following acute traumatic SCI.

Although the effects of hyperglycemia on survival after SCI were understudied, there is a growing body of literature on the detrimental effects of hyperglycemia on survival after more severe TBI. In a retrospective case series in a Level 1 trauma center (*n* = 77), Jeremitsky and colleagues^16^ reported that overall hyperglycemia score and hyperglycemia score for days 3 to 5 post-injury were significantly correlated with mortality after severe TBI (GCS score ≤8). In a retrospective observational study in a Level 1 trauma center (*n* = 429), Liu-DeRyke and associates^17^ documented that patients with TBI who maintained glycemic levels below 160 mg/dL within the first 24 h post-injury showed significantly better survival rates within the initial 30 days from admission to an intensive care unit than their counterparts with glycemic levels of 160 mg/dL or higher. In a prospective cohort study that excluded individuals with prior history of diabetes mellitus (*n* = 220), Kafaki and co-workers^18^ showed that the in-hospital mortality rate in the group of individuals with severe TBI who had hyperglycemia on admission was significantly greater than in the group of patients with glycemia levels below 200 mg/dL (65.8% vs. 23.7%, respectively). In a more recent study analyzing data from a propensity score-matched population (*n* = 1798), Rau and colleagues^19^ reported that individuals with either stress-induced hyperglycemia or diabetic hyperglycemia had a 9.1-fold to 2.3-fold greater odds of death, respectively, than patients with non-diabetic normoglycemia after moderate-to-severe TBI. However, the adjusted mortality was significantly higher in the propensity score-matched patients with stress-induced hyperglycemia, but not in those patients with diabetic hyperglycemia.^19^ Of note, their definition of hyperglycemia was a serum blood concentration ≥200 mg/dL upon arrival in the emergency department.^19^

Overall, the results of this study suggest that persistent hyperglycemia by day 7 post-injury is associated with greater mortality rates after acute traumatic SCI, which is similar to reports in the literature on mortality after moderate or severe TBI. Pragmatically, it remains unclear whether hyperglycemia is a modifiable risk factor of mortality after CNS injuries.^10^ In a recent systematic review and meta-analysis including data on 1066 patients with TBI from 10 randomized clinical trials, Hermanides and associates^20^ concluded that intensive glycemic control did not significantly reduce mortality after TBI. Moreover, further investigations are needed to determine the causes for the higher mortality among individuals with hyperglycemia at day 7 after acute traumatic SCI.

### Hyperglycemia and recovery after CNS injury

The results of this study suggest that hyperglycemia (defined as random glycemic levels >140 mg/dL or >180 mg/dL) is not associated with neurological recovery after acute traumatic SCI, after adjusting for major potential confounders. Among survivors, glycemic levels higher than 140 mg/dL at 24 h post-injury were significantly associated with higher FIM scores at 1 year following acute traumatic SCI. However, persistent hyperglycemia (defined as glycemic levels >140 mg/dL) at day 7 post-injury was significantly associated with lower FIM scores at 6 weeks, at 6 months, and at 1 year after acute traumatic SCI.

Prior experimental studies using animal models provided conflicting results on the potential effects of hyperglycemia on metabolism dysfunction and tissue preservation after SCI. For instance, Sala and colleagues^21^ studied the role of hyperglycemia on the recovery of rats that sustained a traumatic SCI at vertebral level T9 caused by a weight-drop impactor. In their series of experiments, the authors found that: 1) elevated glycemic levels were detected only in the more severely injured animals; 2) pre-injury, overnight fasting did not directly affect lesion volume but caused mild acidosis that had a minor neuroprotective effect; and 3) moderate hyperglycemia induced within 1 h after SCI did not significantly influence the lesion volume.^21^

On the other hand, Kobayakawa and associates^22^ documented that hyperglycemia induced in mice by intraperitoneal injection of streptozotocin was associated with enhanced pro-inflammatory reactivity in the microglial cells (as assessed using “*in vivo*” cell type–specific gene expression analysis with flow cytometry), greater apoptosis activity within the spinal cord, smaller area of preserved spinal cord tissue, and poorer behavioral test score after contusion injury at the T10 level when compared with sham mice. More recently, Chen and co-workers^23^ reported that rats with hyperglycemia (provoked by intraperitoneal injection of streptozotocin) and weight-drop-induced SCI at the T10 level developed prominent endoplasmic reticulum stress, neuronal apoptosis, and increased permeability of the blood–spinal cord barrier within the spinal cord when compared with control groups (i.e., sham group and SCI-alone group).

Similar to the present study, prior clinical investigations demonstrated that hyperglycemia is a common finding in the hyperacute stage after traumatic SCI.^21,22^ However, little is known about the effects of hyperglycemia on neurological and functional recovery after acute traumatic SCI. In a recent retrospective cohort study, Kobayakawa and associates^22^ reported that individuals with acute traumatic SCI who presented hyperglycemia (defined as glycemic level ≥126 mg/dL on admission) showed a greater proportion of poor outcome (American Spinal Injury Association [ASIA] Impairment Scale level A, B, or C), lower ASIA motor scores, and lower Spinal Cord Independence Measure (SCIM) scores at discharge than individuals with normoglycemia post-SCI. However, their data analysis did not control for other potential confounders that can influence that apparent correlation between hyperglycemia and outcomes after acute traumatic SCI.

First, individuals' sex and age can conceivably influence hyperglycemia and outcomes after acute traumatic SCI. Among able-bodied individuals, Basu and co-workers^24^ documented that age and sex can influence insulin secretion, insulin action, hepatic insulin extraction, and glucose effectiveness, which can result in substantial differences in the regulation of glucose metabolism. Also, Li and colleagues^25^ suggested that there are sex-related discrepancies in the glucose metabolism that cannot be only explained by the differences of body composition between females and males. Although sex-related differences in neurological and functional recovery after acute traumatic SCI have been reported previously, the potential effects of individuals' sex on outcomes after SCI remains a matter of debate.^26–28^ Older age at SCI onset has consistently been correlated with higher post-injury mortality rates. Although a few prior studies have reported that older age at the time of injury could be associated with poorer outcomes after traumatic SCI, the most recent investigations using models adjusted for major potential confounders have challenged that chronologic age is an independent factor for less favorable outcomes following acute traumatic SCI.^29–33^

Second, the frequency of concomitant TBI among individuals with acute traumatic SCI was estimated to be 32.5% (95% confidence interval [CI]: 10.8–59.3%) in a recent meta-analysis.^34^ Moreover, hyperglycemia post-injury has a negative impact on neurological and functional recovery after moderate-to-severe TBI, which justifies consideration of concomitant TBI as a major potential confounder in any data analysis focused on recovery after acute traumatic SCI.^10,17,20^

Third, the kidneys play the foremost role in glucose and insulin metabolism, and current knowledge suggests kidneys are intimately involved in the development, maintenance, and resolution of hyperglycemia in critically ill patients.^35^ Therefore, data analyses on the potential effects of hyperglycemia on neurological recovery following acute traumatic SCI ought to account renal function as a potential confounder.

Fourth, the severity and level of SCI are well-recognized prognostic factors for recovery after traumatic SCI and, hence, both factors should be considered in the data analyses as potential confounders.^36^ Further, Torbati and associates^37^ reported that at an acute care spine trauma in Iran individuals with more severe SCI had higher glycemic levels on admission.

Finally, some pharmacological therapies may have an effect on the glycemic levels and outcomes. In this study, glycemic levels higher than 140 mg/dL at 24 h post-injury were significantly associated with higher FIM scores at 1 year following SCI. Although the actual reasons for these findings remain unclear, one may speculate that the infusion of MPSS could be associated with temporary hyperglycemia at 24 h and better functional recovery.^38^

Bracken and colleagues^12^ documented that infusion of MPSS was associated with improved FIM scores at 6 months after injury. On the other hand, in this study those individuals who had persistently elevated glycemic levels within the first week after SCI showed less favorable functional recovery within the first year after SCI. Although a definite explanation cannot be determined, one may wonder whether this group of individuals with persistently elevated glycemic levels within the first week after SCI had a pre-accident glucose metabolism disorder such as diabetes mellitus or pre-diabetes. In a recent combined experimental animal and clinical study, Park and associates^39^ examined the effects of pre-injury chronic hyperglycemia on inflammatory markers and functional recovery after traumatic SCI. In the clinical part of the study, hemoglobin A1c (HbA1c) at admission was associated with poor functional recovery, and those patients with chronic hyperglycemia (defined as HbA1c ≥6.5%) had high concentrations of inflammatory biomarkers (IL-6 and IL-8) in the cerebrospinal fluid following SCI.^39^ Similarly, pre-injury chronic hyperglycemia in rats has been shown to be associated with increased inflammatory responses and oxygen-free radicals in the spinal cord and blood, and consequently resulted in greater lesion volume, less spared tissue, and poorer functional recovery.^39^

Overall, few prior pre-clinical and clinical studies have suggested that hyperglycemia in the hyperacute post-injury stage could be associated with poorer outcomes after acute traumatic SCI. However, the heterogeneity of the SCI population and many potential major confounders represent methodological challenges when carrying out clinical studies in this field.^40^ In the present study it was attempted to address some of those challenges and, after adjusting data analyses for major potential confounders, hyperglycemia within the first week post-injury was found not to be associated with neurological recovery in the sub-acute and chronic stages after traumatic SCI. Nonetheless, hyperglycemia at day 7 post-injury was significantly correlated with greater degree of disability as assessed using the FIM score in the sub-acute and chronic stages following traumatic SCI.

### Study limitations

This retrospective cohort study analyzed a large data set from the NASCIS-3 that included data on several major potential confounders. However, there are limitations that should be taken into account when interpreting and applying the results of this study. First, the retrospective nature of this study precluded collection of other potential confounders. For instance, pre-existing medical co-morbidities could have an impact not only on survival, but also on functional recovery after acute traumatic SCI.^33^ Data on pre-injury diabetes mellitus as a potential confounder were missing in the NASCIS-3 database, even though the estimated prevalence of 4.9% in the United States in 1990 was significantly lower than the estimated global prevalence of 9.3% for 2019.^10,11,41^ Also, polypharmacy and rehabilitation intensity can have important consequences on the neurological and functional outcomes following acute traumatic SCI.^42,43^

Second, the participants in the NASCIS-3 were recruited from 1991 to 1995, when some aspects of pre-hospital, acute spine care, and rehabilitation practices could have been discrepant from the current guidelines. For example, maintenance of mean arterial blood pressure greater than 85 mm Hg, pre-operative evaluation preferentially using magnetic resonance imaging, and early spinal cord decompression have more recently become the best practices in the initial management of patients with acute traumatic SCI.^5,8,44^ Finally, all participants in the NASCIS-3 received a drug that could potentially have a neuroprotective effect in individuals with acute traumatic SCI.

## Conclusion

The results of this retrospective cohort study suggest that hyperglycemia commonly occurs among individuals with acute traumatic SCI. Although hyperglycemia within the first 48 h post-injury did not adversely affect survival after SCI, persistent hyperglycemia at day 7 post-injury was significantly associated with reduced survival rates within the first year following acute traumatic SCI. Additionally, hyperglycemia post-injury did not influence neurological recovery within the first year after acute traumatic SCI. Although hyperglycemia within the initial 48 h post-injury did not negatively affect functional recovery after SCI, hyperglycemia at day 7 post-injury was significantly correlated with greater degree of disability as assessed using the FIM score within the first year following acute traumatic SCI. Those results endorse the current practice guidelines that recommend glycemic control during management of patients with acute traumatic SCI. Nevertheless, the optimal glycemic target in the management of patients with acute traumatic SCI remains to be determined, whereas hypoglycemia should be prevented at all times given its risk for irreversible CNS injury.^9,14,45^

## Supplementary Material

Supplemental data

Supplemental data

Supplemental data

Supplemental data

## Supplementary Material

Supplementary Table SA1

Supplementary Table SA2

Supplementary Table SA3

Supplementary Table SA4

Supplementary Table SB1

Supplementary Table SB2

Supplementary Table SB3

Supplementary Table SB4

## Abbreviations Used

ASIA: American Spinal Injury Association
CI: confidence interval
CNS: central nervous system
FIM: Functional Independence Measure
GCS: Glasgow Coma Scale
HbA1c: hemoglobin A1c
IL: interleukin
MPSS: methylprednisolone
NASCIS-3: Third National Spinal Cord Injury Study
SCI: spinal cord injury
SCIM: Spinal Cord Independence Measure
TBI: traumatic brain injury
TNF-α: tumor necrosis factor alpha
