# Supplementary material for: Effects on Outcomes of Hyperglycemia in the Hyperacute Stage after Acute Traumatic Spinal Cord Injury
Source: Neurotrauma Rep. 2021 Jan 19;2(1):14–24. doi: 10.1089/neur.2020.0042 (PMC8240828; doi:10.1089/neur.2020.0042)
Supplement: Supplemental data [file Supp_TableSA1-S3.docx]

**Table A.1.** Results of the regression analyses on the potential effects of hyperglycemia (threshold: > 140 mg/dL) at 24 hours after the SCI onset to degree of impairment after adjusting the model for the individuals’ age and sex, severity and level of SCI, NASCIS-3 trial drug protocol, Glasgow Coma Score on admission, and serum creatinine concentration (that was collected at the same time of the glycemic test).

| **Dependent variable** |  | **R-square** | **F value** | **P value** |
| --- | --- | --- | --- | --- |
| Motor score at 6 weeks | Model | 0.597 | 55.24 | <0.0001 |
|  | Hyperglycemia at 24 hrs |  | 0.67 | 0.4150 |
| Sensory score at 6 weeks | Model | 0.594 | 53.96 | <0.0001 |
|  | Hyperglycemia at 24 hrs |  | 0.00 | 0.9791 |
| Pain score at 6 weeks | Model | 0.548 | 46.55 | <0.0001 |
|  | Hyperglycemia at 24 hrs |  | 1.55 | 0.2142 |
| Motor score at 6 months | Model | 0.520 | 39.56 | <0.0001 |
|  | Hyperglycemia at 24 hrs |  | 0.80 | 0.3724 |
| Sensory score at 6 months | Model | 0.561 | 46.42 | <0.0001 |
|  | Hyperglycemia at 24 hrs |  | 0.08 | 0.7711 |
| Pain score at 6 months | Model | 0.494 | 36.46 | <0.0001 |
|  | Hyperglycemia at 24 hrs |  | 2.62 | 0.1067 |
| Motor score 1 year | Model | 0.474 | 31.90 | <0.0001 |
|  | Hyperglycemia at 24 hrs |  | 0.78 | 0.3774 |
| Sensory score at 1 year | Model | 0.526 | 39.26 | <0.0001 |
|  | Hyperglycemia at 24 hrs |  | 0.72 | 0.3979 |
| Pain score at 1 year | Model | 0.494 | 35.25 | <0.0001 |
|  | Hyperglycemia at 24 hrs |  | 2.29 | 0.1315 |

**Table A.2.** Results of the regression analyses on the potential effects of hyperglycemia (threshold: > 140 mg/dL) at 48 hours after the SCI onset to degree of impairment after adjusting the model for the individuals’ age and sex, severity and level of SCI, NASCIS-3 trial drug protocol, Glasgow Coma Score on admission, and serum creatinine concentration (that was collected at the same time of the glycemic test).

| **Dependent variable** |  | **R-square** | **F value** | **P value** |
| --- | --- | --- | --- | --- |
| Motor score at 6 weeks | Model | 0.596 | 55.05 | <0.0001 |
|  | Hyperglycemia at 48 hrs |  | 0.00 | 0.9827 |
| Sensory score at 6 weeks | Model | 0.594 | 53.97 | <0.0001 |
|  | Hyperglycemia at 48 hrs |  | 0.06 | 0.8012 |
| Pain score at 6 weeks | Model | 0.546 | 46.19 | <0.0001 |
|  | Hyperglycemia at 48 hrs |  | 0.07 | 0.7925 |
| Motor score at 6 months | Model | 0.596 | 55.05 | <0.0001 |
|  | Hyperglycemia at 48 hrs |  | 0.00 | 0.9827 |
| Sensory score at 6 months | Model | 0.594 | 53.97 | <0.0001 |
|  | Hyperglycemia at 48 hrs |  | 0.06 | 0.8012 |
| Pain score at 6 month | Model | 0.546 | 46.19 | <0.0001 |
|  | Hyperglycemia at 48 hrs |  | 0.07 | 0.7925 |
| Motor score at 1 year | Model | 0.472 | 31.73 | <0.0001 |
|  | Hyperglycemia at 48 hrs |  | 0.00 | 0.9802 |
| Sensory score at 1 year | Model | 0.525 | 39.11 | <0.0001 |
|  | Hyperglycemia at 48 hrs |  | 0.06 | 0.8038 |
| Pain score at 1 year | Model | 0.491 | 34.82 | <0.0001 |
|  | Hyperglycemia at 48 hrs |  | 0.29 | 0.5935 |

**Table A.3.** Results of the regression analyses on the potential effects of hyperglycemia (threshold: > 140 mg/dL) at day 7 after the SCI onset to degree of impairment after adjusting the model for the individuals’ age and sex, severity and level of SCI, NASCIS-3 trial drug protocol, Glasgow Coma Score on admission, and serum creatinine concentration (that was collected at the same time of the glycemic test).

| **Dependent variable** |  | **R-square** | **F value** | **P value** |
| --- | --- | --- | --- | --- |
| Motor score at 6 weeks | Model | 0.634 | 58.72 | <0.0001 |
|  | Hyperglycemia at day 7 |  | 2.90 | 0.0898 |
| Sensory score at 6 weeks | Model | 0.633 | 57.90 | <0.0001 |
|  | Hyperglycemia at day 7 |  | 0.13 | 0.7172 |
| Pain score at 6 weeks | Model | 0.581 | 48.72 | <0.0001 |
|  | Hyperglycemia at day 7 |  | 0.18 | 0.6702 |
| Motor score at 6 months | Model | 0.510 | 34.67 | <0.0001 |
|  | Hyperglycemia at day 7 |  | 3.25 | 0.0726 |
| Sensory score at 6 months | Model | 0.556 | 41.43 | <0.0001 |
|  | Hyperglycemia at day 7 |  | 0.54 | 0.4650 |
| Pain score at 6 months | Model | 0.493 | 33.15 | <0.0001 |
|  | Hyperglycemia at day 7 |  | 0.00 | 0.9552 |
| Motor score at 1 year | Model | 0.461 | 27.86 | <0.0001 |
|  | Hyperglycemia at day 7 |  | 1.31 | 0.2534 |
| Sensory score at 1 year | Model | 0.514 | 34.35 | <0.0001 |
|  | Hyperglycemia at day 7 |  | 0.13 | 0.7165 |
| Pain score at 1 year | Model | 0.489 | 31.82 | <0.0001 |
|  | Hyperglycemia at day 7 |  | 0.18 | 0.6684 |
