# Supplementary material for: Effects on Outcomes of Hyperglycemia in the Hyperacute Stage after Acute Traumatic Spinal Cord Injury
Source: Neurotrauma Rep. 2021 Jan 19;2(1):14–24. doi: 10.1089/neur.2020.0042 (PMC8240828; doi:10.1089/neur.2020.0042)
Supplement: Supplemental data [file Supp_TableSA4.docx]

**Table A.4.** Results of the regression analyses on the potential effects of hyperglycemia (threshold: > 140 mg/dL) on the degree of disability (as assessed using FIM) after adjusting for motor scores at the same time, individuals’ age and sex, NASCIS-3 trial drug protocol, level of SCI, Glasgow Coma Score on admission, and serum creatinine concentration (that was collected at the same time of the glycemic test).

| **Dependent variable** |  | **R-square** | **F value** | **P value** |
| --- | --- | --- | --- | --- |
| FIM score at 6 weeks | Model | 0.600 | 57.29 | <0.0001 |
|  | Hyperglycemia at 24 hrs |  | 0.66 | 0.4162 |
| FIM score at 6 months | Model | 0.535 | 42.06 | <0.0001 |
|  | Hyperglycemia at 24 hrs |  | 3.72 | 0.0547 |
| FIM score at 1 year | Model | 0.494 | 34.61 | <0.0001 |
|  | Hyperglycemia at 24 hrs * |  | 5.26 | 0.0225 |
| FIM score at 6 weeks | Model | 0.770 | 125.81 | <0.0001 |
|  | Hyperglycemia at 48 hrs |  | 0.04 | 0.8336 |
| FIM score at 6 months | Model | 0.787 | 136.33 | <0.0001 |
|  | Hyperglycemia at 48 hrs |  | 0.04 | 0.8407 |
| FIM score at 1 year | Model | 0.768 | 117.75 | <0.0001 |
|  | Hyperglycemia at 48 hrs |  | 0.02 | 0.8850 |
| FIM score at 6 weeks | Model | 0.754 | 106.43 | <0.0001 |
|  | Hyperglycemia at day 7 ** |  | 7.70 | 0.0059 |
| FIM score at 6 months | Model | 0.788 | 126.64 | <0.0001 |
|  | Hyperglycemia at day 7 ** |  | 9.74 | 0.0020 |
| FIM score at 1 year | Model | 0.773 | 113.68 | <0.0001 |
|  | Hyperglycemia at day 7 ** |  | 8.09 | 0.0048 |

* Positive effect of hyperglycemia; ** Negative effect of hyperglycemia
