# Supplementary material for: Effects on Outcomes of Hyperglycemia in the Hyperacute Stage after Acute Traumatic Spinal Cord Injury
Source: Neurotrauma Rep. 2021 Jan 19;2(1):14–24. doi: 10.1089/neur.2020.0042 (PMC8240828; doi:10.1089/neur.2020.0042)
Supplement: Supplemental data [file Supp_TableSB1-B3.docx]

**Table B.1.** Results of the regression analyses on the potential effects of hyperglycemia (threshold: > 180 mg/dL) at 24 hours after the SCI onset to degree of impairment after adjusting the model for the individuals’ age and sex, severity and level of SCI, NASCIS-3 trial drug protocol, Glasgow Coma Score on admission, and serum creatinine concentration (that was collected at the same time of the glycemic test).

| **Dependent variable** |  | **R-square** | **F value** | **P value** |
| --- | --- | --- | --- | --- |
| Motor score at 6 weeks | Model | 0.596 | 55.07 | <0.0001 |
|  | Hyperglycemia at 24 hrs |  | 0.07 | 0.7933 |
| Sensory score at 6 weeks | Model | 0.594 | 53.96 | <0.0001 |
|  | Hyperglycemia at 24 hrs |  | 0.00 | 0.9661 |
| Pain score at 6 weeks | Model | 0.548 | 46.58 | <0.0001 |
|  | Hyperglycemia at 24 hrs |  | 1.68 | 0.1957 |
| Motor score at 6 months | Model | 0.519 | 39.50 | <0.0001 |
|  | Hyperglycemia at 24 hrs |  | 0.54 | 0.4612 |
| Sensory score at 6 months | Model | 0.561 | 46.45 | <0.0001 |
|  | Hyperglycemia at 24 hrs |  | 0.17 | 0.6797 |
| Pain score at 6 months | Model | 0.493 | 36.25 | <0.0001 |
|  | Hyperglycemia at 24 hrs |  | 1.65 | 0.2002 |
| Motor score 1 year | Model | 0.473 | 31.82 | <0.0001 |
|  | Hyperglycemia at 24 hrs |  | 0.42 | 0.5193 |
| Sensory score at 1 year | Model | 0.525 | 39.11 | <0.0001 |
|  | Hyperglycemia at 24 hrs |  | 0.06 | 0.8139 |
| Pain score at 1 year | Model | 0.492 | 35.02 | <0.0001 |
|  | Hyperglycemia at 24 hrs |  | 1.20 | 0.2747 |

**Table B.2.** Results of the regression analyses on the potential effects of hyperglycemia (threshold: > 180 mg/dL) at 48 hours after the SCI onset to degree of impairment after adjusting the model for the individuals’ age and sex, severity and level of SCI, NASCIS-3 trial drug protocol, Glasgow Coma Score on admission, and serum creatinine concentration (that was collected at the same time of the glycemic test).

| **Dependent variable** |  | **R-square** | **F value** | **P value** |
| --- | --- | --- | --- | --- |
| Motor score at 6 weeks | Model | 0.600 | 55.90 | <0.0001 |
|  | Hyperglycemia at 48 hrs |  | 3.07 | 0.0809 |
| Sensory score at 6 weeks | Model | 0.594 | 53.97 | <0.0001 |
|  | Hyperglycemia at 48 hrs |  | 0.05 | 0.8262 |
| Pain score at 6 weeks | Model | 0.548 | 46.59 | <0.0001 |
|  | Hyperglycemia at 48 hrs |  | 1.71 | 0.1915 |
| Motor score at 6 months | Model | 0.600 | 55.90 | <0.0001 |
|  | Hyperglycemia at 48 hrs |  | 3.07 | 0.0809 |
| Sensory score at 6 months | Model | 0.594 | 53.97 | <0.0001 |
|  | Hyperglycemia at 48 hrs |  | 0.05 | 0.8262 |
| Pain score at 6 month | Model | 0.548 | 46.59 | <0.0001 |
|  | Hyperglycemia at 48 hrs |  | 1.71 | 0.1915 |
| Motor score at 1 year | Model | 0.477 | 32.31 | <0.0001 |
|  | Hyperglycemia at 48 hrs |  | 2.76 | 0.0974 |
| Sensory score at 1 year | Model | 0.525 | 39.10 | <0.0001 |
|  | Hyperglycemia at 48 hrs |  | 0.03 | 0.8690 |
| Pain score at 1 year | Model | 0.493 | 35.07 | <0.0001 |
|  | Hyperglycemia at 48 hrs |  | 1.46 | 0.2281 |

**Table B.3.** Results of the regression analyses on the potential effects of hyperglycemia (threshold: > 180 mg/dL) at day 7 after the SCI onset to degree of impairment after adjusting the model for the individuals’ age and sex, severity and level of SCI, NASCIS-3 trial drug protocol, Glasgow Coma Score on admission, and serum creatinine concentration (that was collected at the same time of the glycemic test).

| **Dependent variable** |  | **R-square** | **F value** | **P value** |
| --- | --- | --- | --- | --- |
| Motor score at 6 weeks | Model | 0.633 | 58.44 | <0.0001 |
|  | Hyperglycemia at day 7 |  | 1.98 | 0.1605 |
| Sensory score at 6 weeks | Model | 0.633 | 57.92 | <0.0001 |
|  | Hyperglycemia at day 7 |  | 0.19 | 0.6645 |
| Pain score at 6 weeks | Model | 0.581 | 48.70 | <0.0001 |
|  | Hyperglycemia at day 7 |  | 0.09 | 0.7588 |
| Motor score at 6 months | Model | 0.509 | 34.62 | <0.0001 |
|  | Hyperglycemia at day 7 |  | 3.01 | 0.0837 |
| Sensory score at 6 months | Model | 0.556 | 41.44 | <0.0001 |
|  | Hyperglycemia at day 7 |  | 0.61 | 0.4368 |
| Pain score at 6 months | Model | 0.493 | 33.15 | <0.0001 |
|  | Hyperglycemia at day 7 |  | 0.02 | 0.8926 |
| Motor score at 1 year | Model | 0.462 | 27.93 | <0.0001 |
|  | Hyperglycemia at day 7 |  | 1.66 | 0.1992 |
| Sensory score at 1 year | Model | 0.515 | 34.44 | <0.0001 |
|  | Hyperglycemia at day 7 |  | 0.52 | 0.4728 |
| Pain score at 1 year | Model | 0.489 | 31.84 | <0.0001 |
|  | Hyperglycemia at day 7 |  | 0.30 | 0.5852 |
