# Supplementary material for: Effects on Outcomes of Hyperglycemia in the Hyperacute Stage after Acute Traumatic Spinal Cord Injury
Source: Neurotrauma Rep. 2021 Jan 19;2(1):14–24. doi: 10.1089/neur.2020.0042 (PMC8240828; doi:10.1089/neur.2020.0042)
Supplement: Supplemental data [file Supp_TableSB4.docx]

**Table B.4.** Results of the regression analyses on the potential effects of hyperglycemia (threshold: > 180 mg/dL) on the degree of disability (as assessed using FIM) after adjusting for motor scores at the same time, individuals’ age and sex, NASCIS-3 trial drug protocol, level of SCI, Glasgow Coma Score on admission, and serum creatinine concentration (that was collected at the same time of the glycemic test).

| **Dependent variable** |  | **R-square** | **F value** | **P value** |
| --- | --- | --- | --- | --- |
| FIM score at 6 weeks | Model | 0.601 | 57.56 | <0.0001 |
|  | Hyperglycemia at 24 hrs |  | 1.63 | 0.2024 |
| FIM score at 6 months | Model | 0.530 | 41.25 | <0.0001 |
|  | Hyperglycemia at 24 hrs |  | 0.31 | 0.5766 |
| FIM score at 1 year | Model | 0.486 | 33.47 | <0.0001 |
|  | Hyperglycemia at 24 hrs |  | 0.00 | 0.9703 |
| FIM score at 6 weeks | Model | 0.771 | 126.19 | <0.0001 |
|  | Hyperglycemia at 48 hrs |  | 0.82 | 0.3655 |
| FIM score at 6 months | Model | 0.787 | 136.66 | <0.0001 |
|  | Hyperglycemia at 48 hrs |  | 0.67 | 0.4123 |
| FIM score at 1 year | Model | 0.768 | 117.86 | <0.0001 |
|  | Hyperglycemia at 48 hrs |  | 0.25 | 0.6149 |
| FIM score at 6 weeks | Model | 0.751 | 104.73 | <0.0001 |
|  | Hyperglycemia at day 7 ** |  | 3.85 | 0.0505 |
| FIM score at 6 months | Model | 0.782 | 122.54 | <0.0001 |
|  | Hyperglycemia at day 7 |  | 1.66 | 0.1991 |
| FIM score at 1 year | Model | 0.767 | 109.87 | <0.0001 |
|  | Hyperglycemia at day 7 |  | 0.10 | 0.7513 |

** Negative effect of hyperglycemia
